# P-940. Is anyone listening? Stewardship Implementation in a Large Health System: Successes and Barriers

**DOI:** 10.1093/ofid/ofaf695.1143

**Published:** 2026-01-11

**Authors:** Melissa Whitman, Erin N Deja, Aldo Barajas-Ochoa, Melissa Godwin, Sangeeta Sastry

**Affiliations:** Virginia Commonwealth University, Richmond, Virginia; VCU Health, Henrico, VA; Virginia Commonwealth University, Richmond, Virginia; VCU Health, Henrico, VA; VCU, Richmond, Virginia

## Abstract

**Background:**

In December 2022, the antimicrobial stewardship program (ASP) at our institution published system-wide internal guidance on the management of uncomplicated gram negative bacteremia. We reviewed data from three hospitals within our system - an 865 bed academic hospital vs two community hospitals (a 466 bed and 32 bed limited access center)- the following year to assess adoption of the recommendations.

**Figure:**
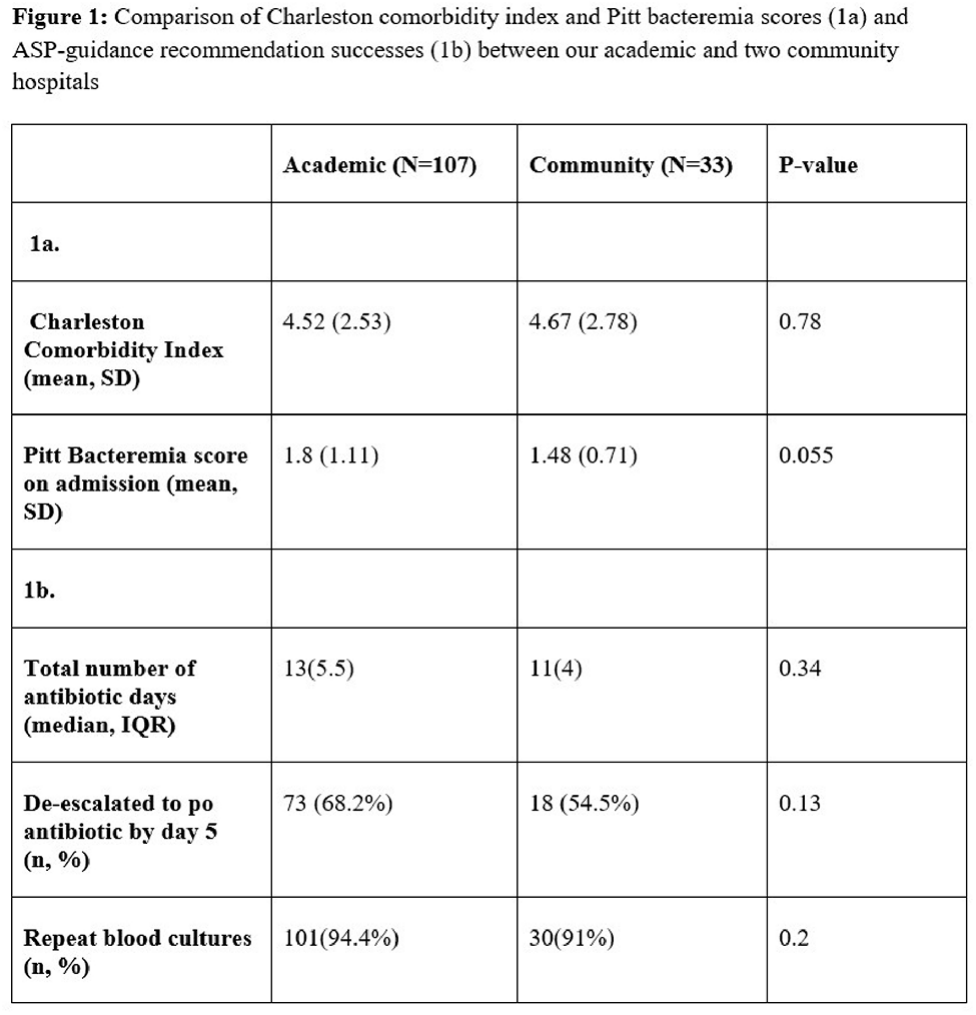
Comparison of Charleston comorbidity index and Pitt bacteremia scores (1a) and ASP-guidance recommendation successes (1b) between our academic and two community hospitals

**Methods:**

We retrospectively reviewed charts of patients with blood cultures positive for an *Enterobacterales* organism between January 1 and December 31, 2023. We included patients who met our pre-defined criteria for uncomplicated bacteremia in the analysis. Summary descriptive statistics were collected and comparisons between the academic and community hospital groups were made using Chi-squared, T-test for independent means, or Welsh T-test as appropriate.

**Results:**

A total of 140 patients met inclusion criteria (107/140 academic, 33/140 community). The primary source of infection was the urinary tract (109/140). The Charleston comorbidity index and Pitt bacteremia score were not different between the two groups (Figure 1a). Median total antibiotic duration was 12 days (p=0.34, Figure 1b). In total, 131/140 patients had surveillance blood cultures drawn (101/107 or 94.4% academic, 30/33 or 90.9% community, p=0.2). By day five, 91/140 patients were de-escalated to PO therapy (73/107 or 68% academic, 18/33 or 55.% community, p=0.13). De-escalation to a PO fluoroquinolone was most frequent across all hospitals (75/91) followed by PO beta-lactams (13/91).

**Conclusion:**

Uptake of ASP recommendations was low at both our academic and community hospitals. De-escalation to PO antibiotics was more frequent at the academic center than in the community, likely as a result of active pharmacy-driven prospective audit and feedback to prompt IV to PO transition, a resource not available at the community hospitals. The dissemination of antimicrobial use guidelines is insufficient for impacting practice change. Future ASP IV to PO initiatives will require active processes such as prospective audit and feedback for maximal adoption.

**Disclosures:**

All Authors: No reported disclosures

